# Anaphylaxis related to avocado ingestion: a case and review

**DOI:** 10.1186/1710-1492-7-12

**Published:** 2011-06-10

**Authors:** Elissa M Abrams, Allan B Becker, Thomas V Gerstner

**Affiliations:** 1 Section of Allergy and Clinical Immunology, Department of Paediatrics and Child Health, University of Manitoba, (685 William Avenue), Winnipeg, (R3A 1S1), Canada

**Keywords:** anaphylaxis, food allergy, avocado

## Abstract

Anaphylaxis to avocado, independent of latex sensitization, has been rarely reported in the literature. This case report describes a 15 year old male who experienced anaphylaxis within half an hour after eating avocado-containing food. Avocado consumption is common in both North America and South America. It is important to consider avocado as a cause of anaphylaxis, even in patients not sensitized to latex.

## Background

Anaphylaxis is a life threatening reaction that occurs in between 0.05 to 2% of the general population [1]. While there have been efforts made to identify and clone the major allergen of avocado [2], Prs a 1, the majority of cases of anaphylaxis to avocado have been described in the context of latex sensitization, otherwise termed "latex-fruit syndrome" [3]. Isolated anaphylaxis to avocado is rarely described in the literature. The following case report describes an individual who developed anaphylaxis after eating guacamole without concurrent clinical reactivity to latex.

## Case presentation

The patient was a 15 year old male who was referred to the allergy clinic for suspected anaphylaxis to avocado. In the summer of 2010, the patient was eating guacamole dip with chips and within a half an hour of eating the guacamole, he developed coughing, wheezing, nasal stuffiness, generalized urticaria and periorbital edema. He did not experience any nausea, vomiting, diarrhea, syncope or loss of consciousness. The patient had eaten the same brand of chips multiple times in the past, but did not remember if he had consumed avocado before.

A family member provided the patient with an antihistamine but the symptoms didn't improve so he went to the emergency department. At the emergency department, intravenous antihistamine and steroid were administered. Within a few hours, the symptoms resolved and the patient was discharged with a script for oral steroid as well as an EpiPen^®^.

The diet of the patient was unrestricted. He had been exposed to latex in the form of balloons multiple times in the past without a reaction. In addition, his diet included other foods that are often included in latex-fruit syndrome (kiwi, banana, potato) [4]. It was unclear if he had previously ingested chestnut.

The patient's past medical history revealed resolved "eczema" to the neck, posterior popliteal and antecubital fossae. There was no history of asthma or allergic rhinitis. The patient reported a history of cold induced hives. The family history included a mother with asthma, an older brother with "environmental allergies" and asthma, and a sister with "environmental allergies" and asthma.

A complete physical examination was unremarkable. The patient underwent epicutaneous testing via prick technique for fresh avocado, commercial avocado extract, fresh and commercial chestnut, and guacamole dip. In addition, an ice cube test was done. Skin testing was positive to fresh avocado (10 mm) and commercial extract (5 mm) and borderline to the guacamole mix (3 mm). Testing to fresh and commercial chestnut was positive to fresh chestnut (10 mm) and commercial chestnut (6 mm). Histamine was 5 mm, and saline control was 2 mm. The ice cube test revealed faint, transient localized urticaria. A latex use test (both finger and full glove) was negative. ImmunoCAP to avocado was positive at 0.9 KU_A_/L.

An oral challenge to chestnut will be considered, should the family be willing, at a later date, to confirm whether a clinical allergy to chestnut exists.

## Conclusions

Avocado is the fruit of the tree Persea americana [5]. IgE-mediated reactions to avocado are well described in the literature, although almost exclusively in the context of latex sensitization. Anaphylaxis to avocado alone is rare. A possible case of isolated anaphylaxis to avocado was described in one multi-center Italian study [6], which examined the frequency of anaphylaxis among food allergic adults. Of 1110 adults with food allergy, 58 had at least one episode of anaphylaxis. Only one of these patients had anaphylaxis to avocado, although it is unclear if they were sensitized to latex.

Sensitization to both avocado and chestnut have also been described, but also almost exclusively in the context of a latex allergy. In 1998 the relevant allergens in chestnut and avocado were described by Diaz-Perales et al [7]. Of the four patients with immediate hypersensitivity to chestnut and avocado, all four also showed associated hypersensitivity to latex.

The prevalence of isolated avocado hypersensitivity, as well as the long-term outcome, is currently unknown.

In a recent study of chestnut allergy, Sanchez-Monge et al examined the difference in allergen sensitization patterns in those who were allergic to chestnut alone, or chestnut with associated latex hypersensitivity [8]. In that study, different patterns of allergen sensitization were present in patients with chestnut allergy with and without latex hypersensitivity (lipid transfer proteins and class I chitinases). One could hypothesize that similar to chestnut, there would be different patterns of allergen sensitization in those patients with avocado allergy, with and without latex hypersensitivity.

## Consent

Written informed consent was obtained from the patient for publication of this case report and any accompanying images. A copy of the written consent is available for review by the Editor-in-Chief of this journal.

## Competing interests

The authors declare that they have no competing interests.

## Authors' contributions

EA and TG diagnosed the case and planned the treatment and medical follow up. EA gathered the patient's history and drafted the manuscript and the subsequent revisions. TG and AB participated in manuscript revision. All authors read and approved the final manuscript.
